# Pain Trends Among American Adults, 2002–2018: Patterns, Disparities, and Correlates

**DOI:** 10.1215/00703370-8977691

**Published:** 2021-04-01

**Authors:** Anna Zajacova, Hanna Grol-Prokopczyk, Zachary Zimmer

**Affiliations:** Department of Sociology, The University of Western Ontario, London, Ontario, Canada; Department of Sociology, University at Buffalo, State University of New York, Buffalo, NY, USA; Department of Family Studies and Gerontology and Global Aging and Community Initiative, Mount Saint Vincent University, Halifax, Nova Scotia, Canada

**Keywords:** Chronic pain, U.S. adults, Trend, Socioeconomic, Health disparities

## Abstract

Determining long-term trends in chronic pain prevalence is critical for evaluating and shaping U.S. health policies, but little research has examined such trends. This study (1) provides estimates of pain trends among U.S. adults across major population groups; (2) tests whether sociodemographic disparities in pain have widened or narrowed over time; and (3) examines socioeconomic, behavioral, psychological, and medical correlates of pain trends. Regression and decomposition analyses of joint, low back, neck, facial/jaw pain, and headache/migraine using the 2002–2018 National Health Interview Survey for adults aged 25–84 (*N* = 441,707) assess the trends and their correlates. We find extensive escalation of pain prevalence in all population subgroups: overall, reports of pain in at least one site increased by 10%, representing an additional 10.5 million adults experiencing pain. Socioeconomic disparities in pain are widening over time, and psychological distress and health behaviors are among the salient correlates of the trends. This study thus comprehensively documents rising pain prevalence among Americans across the adult life span and highlights socioeconomic, behavioral, and psychological factors as important correlates of the trends. Chronic pain is an important dimension of population health, and demographic research should include it when studying health and health disparities.

## Introduction

This study analyzes trends in chronic pain prevalence from 2002 to 2018 among U.S. adults aged 25–84.^1^ We show that pain prevalence—already high at baseline—increased substantially during the study period, with the increase evident in all leading pain sites (joint, low back, neck, facial/jaw pain, and headache/migraine). Although the rise in pain prevalence occurred in nearly all population subgroups, adults at lower socioeconomic levels experienced steeper pain increases, resulting in widening pain disparities by socioeconomic status (SES). We also identify a cluster of salient intermediate and proximal correlates of the pain increases, which include psychological distress, alcohol use, body weight, and arthritis. This study thus provides the first comprehensive portrait of recent pain trends and their individual-level correlates in the U.S. adult population.

Chronic pain is a major public health problem given its high prevalence and costs (Croft et al. 2011). Nationally, the number of people experiencing chronic pain exceeds those affected by heart disease, cancer, and diabetes combined (Institute of Medicine 2011). In 2012, the annual economic cost of pain in the United States was estimated at more than $600 billion (Gaskin and Richard 2012) and has likely increased since then. For individuals, chronic pain is a key determinant of quality of life (Dueñas et al. 2016), healthcare utilization (Song et al. 2016), and disability (Rice et al. 2016). Moreover, pain and pain treatments are linked to the unprecedented up-surge of opioid-related overdoses and deaths among American adults (Ahmad et al. 2018)—a public health crisis in its own right.

Recent decades have seen an “explosion” (Gatchel et al. 2007) of research on chronic pain in the health sciences, epidemiology, and psychology. Demographers, however, have remained largely silent on the topic. For example, *Demography,* one of the highest impact journals in population science, has in its 56-year history published only two articles with “pain” in their title or abstract, neither of which treated pain as their primary topic (Hamilton et al. 2019; Reither et al. 2009). This may be a function of the peculiar status of pain, which until recently was viewed largely as a symptom of other conditions rather than as a condition in itself (Cohen et al. 2013; Raffaeli and Arnaudo 2017). However, there is a growing consensus in the medical literature that chronic pain should be considered a disease in its own right (Siddall 2013; Volkow and McLellan 2016)—as formalized by the inclusion of a “chronic pain” classification in the ICD-11 (Smith et al. 2019)—and that pain’s high population burden necessitates focused interdisciplinary attention (Croft et al. 2011). Our study thus adds a much-needed demographic perspective on pain in the United States.

Assessing the future burden of chronic pain (Interagency Pain Research Coordinating Committee 2018) requires an understanding of recent trends in pain prevalence. Unfortunately, the literature on pain trends is sparse. Scattered studies have focused on pain at specific body sites or in particular clinical or community dwelling populations. For instance, studies have found that adults in North Carolina experienced increasing back pain from 1992 to 2006 (Freburger et al. 2009); nursing home residents reported less chronic pain from 2006 to 2009 (Shen et al. 2015); and non-Hispanic Whites aged 45–64 experienced increases in chronic pain from 1999 to 2013 (Case and Deaton 2015). We are aware of only three U.S. studies using nationally representative samples to examine pain trends. However, two of those (Grol-Prokopczyk 2017; Zimmer and Zajacova 2020) included only older adults, and the third (Nahin et al. 2019) focused on ICD-coded “painful conditions” rather than general chronic pain. All three, nonetheless, reported increasing prevalence. For example, the proportion of adults with at least one “painful health condition” increased from 33% in 1997 to 41% in 2014, a statistically significant and substantively meaningful increase (Nahin et al. 2019).

This limited literature leaves unexplored important topics that we address here. Specifically, we generate up-to-date estimates of pain trends (the most recent published data are from 2014). We include individuals across the adult life span, aged 25–84, while also examining the data across age groups (25–44, 45–64, and 65–84), representing different life course stages and birth cohorts. This is important because older adults report more pain than younger adults (Kennedy et al. 2014; Nahin 2015), and pain correlates may also vary by age (Edwards 2006; Grol-Prokopczyk et al. 2017). We also examine trends by sex, race, and SES. Pain *prevalence* differs substantially across these characteristics: it is higher among women than men (Bartley and Fillingim 2013); higher for adults with lower SES (Jay et al. 2019; Riskowski 2014); and, in most U.S.-based studies, higher among non-Hispanic Whites than among minorities (Kennedy et al. 2014; Nahin 2015). It is therefore reasonable to ask whether pain *trends* also differ across sociodemographic groups. To our knowledge, the single prior study that tested for group heterogeneity in pain trends found no significant differences, albeit only among older adults (Grol-Prokopczyk 2017). The present study formally tests for trend differences to ascertain whether social and demographic disparities in pain are decreasing or increasing over time.

Beyond describing pain trends, it is critical to identify salient social and medical factors associated with the trends. Utilizing the rich set of covariates available in the National Health Interview Survey (NHIS) and linking them to changes in pain over time, our study provides the first comprehensive findings on this topic. Although such correlational analyses cannot establish causality, they provide suggestive insights regarding processes that underlie changes in population pain prevalence and can motivate future in-depth causal analyses of key correlates.

Informed by the WHO health determinants framework (Solar and Irwin 2010) and social determinants of pain models (Craig and Fashler 2013), we conceptualize pain prevalence as a function of a complex web of causation that includes sociodemographic characteristics (which shape exposure to risk and access to resources), intermediate-level health-behavioral and psychological characteristics, and proximate pain-producing medical conditions. Importantly, the WHO framework also posits a critical upstream level comprising the socioeconomic-political context that shapes all individual-level relationships, but available data restrict the scope of our study to individual-level factors. *Socioeconomic factors* closely linked to pain include education (Zajacova et al. 2020), employment status (Fliesser et al. 2017), and economic resources (Riskowski 2014). *Demographic characteristics* include age, sex, race, nativity, and language (Kennedy et al. 2014; Nahin 2015). *Health behaviors/characteristics* shown to impact pain include smoking, alcohol use, body weight, and physical activity (Kennedy et al. 2014; van Hecke et al. 2013). *Psychological factors* linked to pain include depression and psychological well-being (Goosby 2013; Hooten 2016). Finally, proximate *medical conditions* strongly related to pain include arthritis, cancer, diabetes, and respiratory disease (Janevic et al. 2017; Nahin 2015). Admittedly, correlates of pain *trends* may differ from correlates of pain prevalence, and theories of the former are lacking. However, demographic studies have identified similar sociodemographic, intermediate, and proximate determinants of disability and mortality trends (Martin and Schoeni 2014; Montez et al. 2019; Zajacova and Montez 2018).

An admitted challenge in any study of long-term pain trends is that norms surrounding pain reporting may change. Recent popular authors speculate that cultural and institutional developments, driven partly by aggressive marketing of opioid analgesics by pharmaceutical companies, may have led Americans to report pain more readily than in the past (Lembke 2016; Quinones 2015). In our Discussion section, we evaluate relevant evidence and conclude that although reporting differences may play some role, they are unlikely to fully explain our findings.

The present study fills the gaps in knowledge about pain trends and their correlates using 2002–2018 data from the NHIS. We pose three questions central to describing pain and pain trends among American adults. First, what are the aggregate trends in pain prevalence for leading pain sites? Unlike prior studies, which examined only one pain site or used a global pain measure, we provide separate estimates for five specific pain sites as well as for a summary pain index. This generates a more granular portrayal of U.S. pain trends. Second, are the trends similar for major sociodemographic groups, and if not, are pain experiences converging or diverging over time? And third, how do sociodemographic, health-behavioral, psychological, and medical factors correlate with the observed pain trends? These three questions collectively allow us to assess how pain prevalence has evolved in the United States from 2002 to 2018, to describe heterogeneity in the trends across groups, and to identify salient individual-level factors linked to the trends.

## Methods

### Data

We use the 2002–2018 NHIS data, harmonized by IPUMS (Blewett et al. 2019). The NHIS is an ongoing cross-sectional, nationally representative survey of the noninstitutionalized population in the United States. It is the best available source of data for this study because it includes adults of all ages, multiple questions about site-specific pain that remain consistent over time, a large set of relevant covariates, ongoing data collection that yields up-to-date estimates, and a large sample size that permits subgroup analyses. All variables needed for our analyses have been collected consistently since 2002; the most recent wave available at the time of writing is from 2018.

The analytic sample is defined as “sample adult” women and men aged 25–84 who were interviewed in a survey wave between 2002 and 2018. The “sample adult” is a random subsample of about 43% of all adult NHIS respondents that was administered the detailed health measures we utilize. The lower age boundary was chosen to minimize the proportion of respondents who were enrolled in a postsecondary educational institution (National Center for Education Statistics 2018) given that their social status information (educational attainment, employment status, and income) remains to be established. The upper age boundary is set at 84 because NHIS respondents’ ages are top coded at 85, and thus 85 encompasses a wide range of actual respondent ages. From the total 443,237 respondents, we excluded 1,530 (0.35%) who had the highest amount of missing independent variables (0.19%) or had missing pain information (0.16%), yielding an analytic sample size of 441,707. The annual sample sizes vary from 19,040 in 2008 to 32,149 in 2014.

### Measures

#### Pain

The NHIS core questionnaire includes questions about pain in five body sites, representing the most common and/or disabling types of pain (Rice et al. 2016). Four questions followed this prompt: “During the past three months, did you have [low back pain, neck pain, severe headache or migraine, or facial or jaw ache or pain]?” The fifth pain indicator (joint pain) was collected with two linked questions. First, respondents were asked whether they had “any symptoms of pain, aching, or stiffness in or around a joint.” Respondents who answered affirmatively were then asked whether the onset was at least three months prior. We used a positive response to this follow-up question as an indicator of chronic joint pain so that all pain measures in this study capture chronic pain occurring over the last three months. The wording of the pain questions differs slightly: joint pain is described as “lasting at least 3 months,” whereas the other sites refer to pain “during the last 3 months.” Nonetheless, as shown later, joint pain is one of the most commonly reported pain sites, and findings were broadly similar across all sites. We also created a measure for “any pain” in which those who responded affirmatively to any of the five pain sites were coded as having pain.

Correlations among the pain sites are moderate, ranging from *r* = .15 between headache/migraine and joint pain to *r* = .39 between low back and neck pain (tetrachoric *r* = .28 and .66, respectively). These results fit with the knowledge that most people will experience pain in multiple sites (Carnes 2011), but they also indicate that each pain measure contributes independent information about respondents’ pain status and can meaningfully be analyzed either separately or jointly.

#### Time

The date of the interview is the key predictor. The NHIS provides information about the month and year of the interview. We created a measure of continuous time, normalized to have a 0 to 1 range, using the formula $\text{continuous time }\;\text{=}\;\left[(\text{ year }-2002)+\frac{\text{ month }-1}{12}\right]/17$. As a result, a one-unit change in the trend coefficient estimated in regression models can be interpreted as the change in pain level from the start (January 2002) to the end (December 2018) of the observation period; that is, the coefficient captures the change across the 17-year period.

#### Covariates

We include covariates that are consistent with the social determinants framework and its chain of causation from demographic and social factors, through health behaviors and psychological distress, to chronic conditions. Age is treated in two ways. First, age in single years is included in all models as a continuous covariate. Second, the sample is stratified into three 20-year age groups: 25–44, 45–64, and 65–84. (The age stratified models also control for age, as appropriate for the 20 year age spans of each group.) Sex is coded with male as reference. Race/ethnicity categories are non-Hispanic White (reference), non-Hispanic Black, Hispanic, and other. Region of residence is Northeast (reference), Midwest, South, and West. We also control for information provided by proxy interview respondents rather than the target individual (reference). Foreign-born status (U.S.-born as the reference) and interviews conducted in a language other than English (English as the reference) are included because immigrant status and language of interview may impact pain experience and/or reporting (Nahin 2015; Viruell-Fuentes et al. 2011).

Two measures of social ties are included. Marital status is categorized as married or cohabiting (reference) versus not married. The presence of children—own, step-, or adopted—currently residing in the household is dichotomous, with no children as the reference.

We include several covariates that measure SES. Educational attainment is categorized as less than high school or a GED, high school diploma, some college or associate degree, and bachelor’s degree or more (reference). GED is grouped with “less than high school” because prior studies found that the health of GED recipients is more comparable to that of high school dropouts than graduates (Zajacova and Montez 2017a). “Some college” is retained as a separate category because this heterogeneous group differs from both high school and college graduates in important ways (Zajacova et al. 2012), including specifically in pain prevalence (Zajacova et al. 2020). Economic well-being is captured with four indicators. First, current employment status is coded as employed (reference) versus not. Second, we include information about employment one year prior to the interview: respondent worked all 12 months (reference), only a part of the year, or not at all. Third, we control for family income-to-poverty ratio calculated by the NHIS. The reported total family income is compared with the year-specific U.S. Census poverty threshold based on family size and the number of children under 18. This adjustment means the family income controls for household composition and for inflation. We refer to this variable as “income” for parsimony and categorize it as more than 4 times the poverty threshold (reference), 2–3.9 times the threshold, 1–1.9 times the threshold, and below the poverty threshold. Fourth, homeownership captures a longer term economic resource and is categorized as homeowner (reference) versus not.

Health behaviors include smoking, alcohol use, body mass index (BMI), and physical activity. Smoking is categorized as never (reference), former, and current. Alcohol use is coded as never, former, current moderate (reference), and excessive current use. The latter is defined as any binge use in the past year (5 or more drinks per day; since 2014, this question was altered in the NHIS for females to 4 or more drinks) or heavy use (8 or more drinks per week for women and 15 or more for men) (Esser et al. 2014). BMI, conceptualized as a measure of long-term dietary behaviors, was calculated by the NHIS from self-reported height and weight and is included in models as a continuous covariate. Physical activity is a dichotomous measure capturing whether a respondent met federal guidelines for physical activity (reference) or not. The threshold to meet the guidelines is 150 minutes of moderate activity or 75 minutes of vigorous exercise per week (U.S. Department of Health and Human Services 2018). Health conditions were assessed in the NHIS using this prompt: “Have you ever been told by a doctor or other health professional that you had [this condition]?” The conditions comprise respiratory disease (COPD or chronic bronchitis), heart disease, arthritis, cancer, diabetes, hypertension, kidney disease, liver condition, and stroke. Finally, the Kessler Scale (K6), which measures psychological distress in the past month (Kessler et al. 2002), is included as a continuous covariate ranging from 0 to 24.

### Approach

The analysis comprised five steps. We used data collected continually from 2002 to 2018, with two exceptions: the descriptive statistics in Step 1 used only 2002 and 2018 data, and the decomposition analysis in Step 5 used only 2002–2004 and 2016–2018 data. Extensive robustness checks were conducted and are summarized in the online appendix.

In Step 1, we summarized pain prevalence and population characteristics in the first and last year of the study period --2002 and 2018. For Table 1, we estimated the prevalence and age-standardized prevalence of pain at each site and “any pain” in 2002 and in 2018, and tested whether the difference was statistically significant using design-adjusted chi-square-alternative *F* tests. The age standardization was based on the 2010 U.S. population age structure. We also calculated the relative change in pain prevalence, defined as (*pain*_20l8_ – *pain*_2002_)/*pain*_2002_, and the absolute percentage point change. The target population characteristics in 2002 and 2018 are summarized in Table 2; the table also shows the *p* value associated with tests of differences in the distribution of each variable between these two years using design-adjusted Wald tests for continuous variables and *F* tests for categorical variables.

**Fig. 1:**
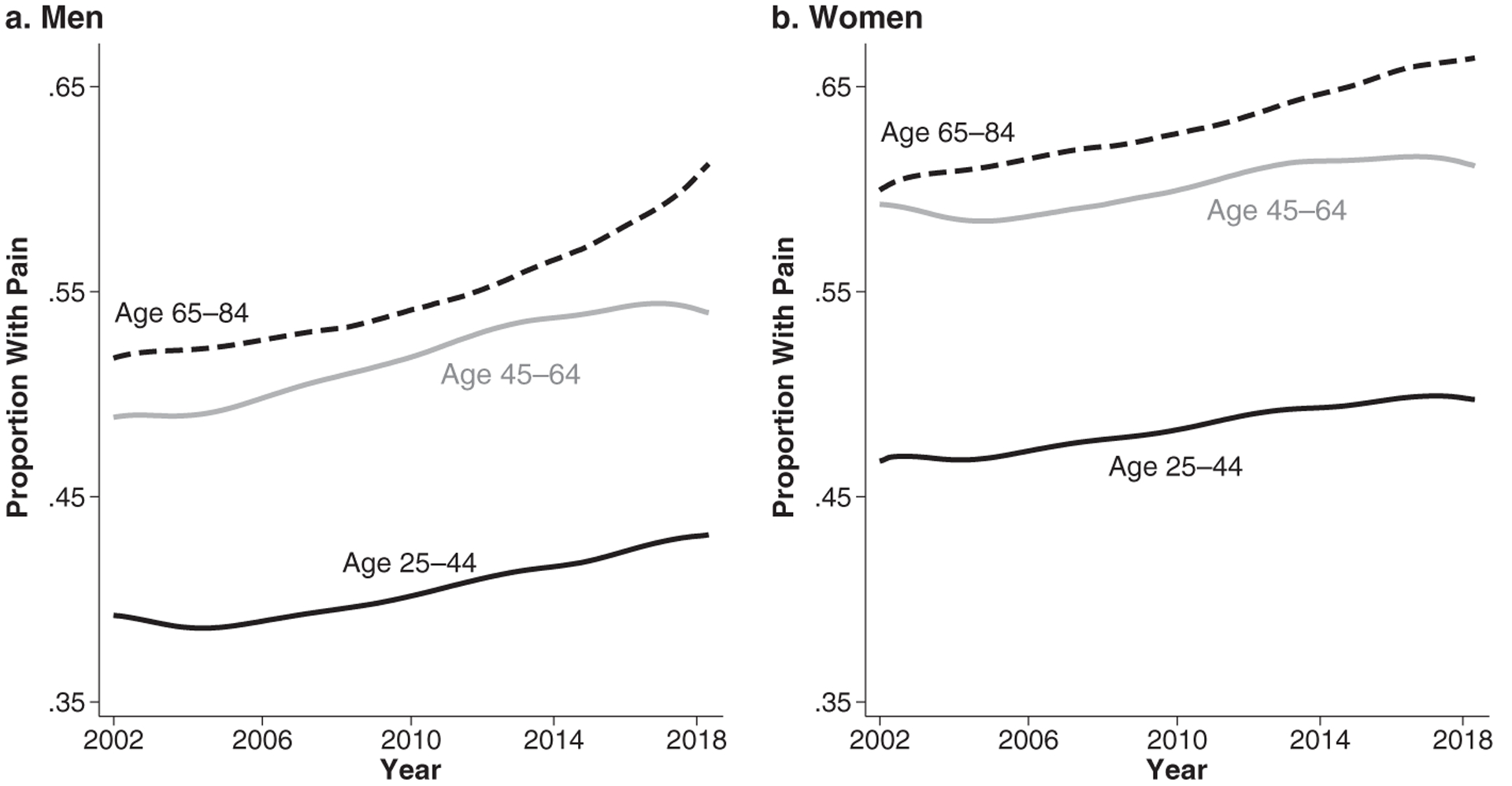
Pain trends 2002–2018 for U.S. adults ages 25–84. Results from a semiparametric age- and sex-stratified, demographics-adjusted logistic model of “any pain.”

Next, we established the functional form of the pain trend in Step 2. This step was important to determine the most parsimonious specification for the time variable. We estimated a series of age-adjusted models of pain with a flexibly specified time trend. These were semiparametric partial-linear models of the form *P*_*i*_ = α + *f* (*t*_*i*_) + γ *x*_*i*_, estimated using the *plreg* command in Stata (Lokshin 2006). Here, *P*_*i*_ is the presence of pain (“any pain” = 1), *x*_*i*_ is age, and *t*_*i*_ captures the date of interview as specified in the Measures section. The smooth function of time *f* (*t*_*i*_) was estimated by the *lowess* procedure in Stata (Cleveland 1979). This model allowed us to capture the time trend nonparametrically while additively including additional variables, such as demographics. The results are plotted as line graphs so the detailed but smoothed shape of the trend can be observed (Figure 1).

In Step 3, we estimated the direction and magnitude of changes in pain over time in demographics-adjusted models for the full sample and major population subgroups. We estimated age-adjusted logistic regression models of each pain measure of the form Logit (*P*_*i*_) = α + β *t*_*i*_ + γ *x*_*i*_, where *P*_*i*_ is the presence of pain, *x*_*i*_ is age, and *t*_*i*_ captures the date of interview. The key coefficient β shows the change in the logit of pain over the observation period (as explained earlier, we coded the date of interview to range from 0 to 1 for this purpose). We estimated demographics-adjusted logit models for “any pain” and each individual pain measure for the total sample, as well as for major population subgroups. The results are shown in Table 3.

For Step 4, we tested whether the pain trends differ statistically across population subgroups. We estimated logistic models of each pain indicator as in the prior step, but instead of stratifying, we included trend by group interactions. For example, to test whether the pain trend differs by sex, we estimated a model of the form Logit(*P_i_*) = α + β*t*_*i*_ + δ *f*_*i*_ + *τ*(*t*_*i*_ *× f*_*i*_) + γ *x*_*i*_, where *f*_*i*_ is a binary indicator of gender (*f*_*i*_ = 0 indicates male; *f*_*i*_ = 1 indicates female), and τ is the coefficient for the interaction between gender and time. In Table 3, we bolded the respective coefficient where differences in trends were statistically significant at *p* < .05.

Finally, in Step 5, we explored factors that correlate with the pain trends. We followed a four-pronged approach to complete this analytically nontrivial task because no single method or model provided a full answer.

First, we examined the role of each covariate independently. This step was important because we consider covariates at different levels of the “chain of causation.” Thus, proximate mechanisms (e.g., medical conditions) may attenuate the role of intermediate mechanisms (e.g., health behaviors), which in turn could attenuate the role of socioeconomic characteristics such as education and income. We estimated pairs of nested models of “any pain.” One model estimated pain as a function of demographics as in Table 3, row 1. The second model added a single covariate and we calculated the percentage change in the log odds of the pain trend coefficient. In Table 4, we list the coefficients in the order that they attenuate or increase the trend coefficient the most; we also show the percentage change in the pain trend coefficient. This approach is widely used in the social sciences to understand the “explanatory” role of covariates on the effect of another predictor. Here, it shows which covariates may be individually salient to the pain trends. However, it also has a major limitation: the models are potentially misspecified because we omit from them other covariates that also significantly impact pain; that is, the results are necessarily biased by omitted variables that are correlated with both the included single covariate and the trend.

Second, we estimated fully adjusted models of pain (Table 5) to observe the pain trend after accounting for all covariates. This model shows what the pain trends would have been if none of the variables’ distributions or effects on pain changed over time. It also shows the average effect of each covariate on pain prevalence. However, such additive models assume that the effect of all variables on pain does not change over time; we therefore next relax this assumption.

Third, we estimated fully adjusted models where each covariate was interacted with time. This model allows us to observe which covariates’ association with pain varies significantly over time (with the caveat that we are allowing only linear change over time while the actual changes might be nonlinear). For parsimony, Table 5 indicates the direction and *p* value of only statistically significant interactions beside the pertinent covariate.

Fourth, Table 6 summarizes findings from the counterfactual Oaxaca-Blinder nonlinear decomposition, which quantifies how much of the difference in pain prevalence between the beginning and end of the observation period is due to different population characteristics (compositional changes) or different relationships between the characteristics and pain (coefficient changes; Blinder 1973; Oaxaca 1973). Conceptually, the observed difference in pain prevalence ${\bar{y}}_{l}-{\bar{y}}_{e}$, where ${\bar{y}}_{l}$ is the mean pain level late in the observation period and ${\bar{y}}_{e}$ is the mean pain level early in the observation period, is defined as ${\bar{y}}_{l}-{\bar{y}}_{e}=F\left({\text{X}}_{l}{\hat{\beta }}_{l}\right)-F\left({\text{X}}_{e}{\hat{\beta }}_{e}\right)$, where the **X**_*l*_, and **X**_*e*_ are matrices of observed covariates late and early in the observation period, respectively. Their associated vectors of $\hat{\beta }$ s are estimated with the logit model, and *F*() is the cumulative distribution function of the logistic distribution. We added and subtracted $F\left({\text{X}}_{e}{\hat{\beta }}_{l}\right)$ to obtain: ${\bar{y}}_{l}-{\bar{y}}_{e}=\left[F\left({\text{X}}_{l}{\hat{\beta }}_{l}\right)-F\left({\text{X}}_{e}{\hat{\beta }}_{l}\right)\right]+\left[F\left({\text{X}}_{e}{\hat{\beta }}_{l}\right)-F\left({\text{X}}_{e}{\hat{\beta }}_{e}\right)\right]$. The first bracket captures the difference between the two groups due to the differences in characteristics, whereas the second bracket captures the part due to differences in coefficients. Because available decomposition approaches rely on the comparison of two groups, we pooled observations collected in the first three years of the observation period (2002–2004) and the most recent three years (2016–2018). We used the *mvdcmp* extension in Stata for decomposition (Powers et al. 2011), combined with the new utility for grouping individual covariates for detailed decomposition, *mvdcmpgroup* (D. Powers, personal communication, February 8, 2020). The effects for categorical variables in this approach are normalized as deviations from a grand mean, which enables calculation of effects for all levels and yields results that are the same regardless of which level is the omitted reference category (Jann 2008).

Overall missingness in the NHIS 2002–2018 data is low. In our analytic sample defined earlier, half of the variables had no missing cases, 13 variables had less than 1% of cases missing, 4 variables had less than 4% missing, and only physical activity (5.4% missing) and family income (13.5% missing) had higher amounts of missingness. Respondents who were older, were female, needed a proxy to complete the interview, resided in the Northeast, and/or had lower SES were more likely to be missing information on select variables than respondents who were younger, male, higher-SES, and/or who resided outside the Northeast. To deal with missingness, we used multiple imputation (MI) via chained equations (Royston and White 2011) for the seven variables with the highest degree of missingness. We created 10 imputed data sets and used Rubin’s rules for combining results in regression models (Rubin 1987). We used a single, randomly selected, multiply imputed data set in the decomposition analysis. We also preprocessed the data via single imputation of select variables. For variables with less than 1% missingness and a clear mode comprising more than 80% of observations, we imputed the mode. For variables with up to 0.5% missingness but a less clear mode, we employed single imputation using all available nonmissing variables. The preprocessing yielded stable and replicable MI results with satisfactory diagnostics.

All regression analyses consider the complex sampling structure of the NHIS. Sampling weights were adjusted for pooling across multiple years (National Center for Health Statistics 2017), and variance adjustment was based on Taylor series linear approximation (Lumley 2004). The analyses were estimated in Stata 15.1 (StataCorp 2017).

## Results

Table 1 shows the weighted crude and age-standardized prevalence of each pain site and “any pain” in the U.S. population aged 25–84 in 2002 and 2018. Prevalence of pain in each body site increased. Correspondingly, so did the prevalence of “any pain,” which increased from 49% to 54%—a change of approximately 10% over time in relative terms. The steepest increases occurred for the highest prevalence pain sites, especially joint pain, which increased by 21% over the 17-year period, and for low back and neck pain (15% and 16% increase, respectively). Facial/jaw pain increased by 13%, and headache/migraine prevalence increased by 5%. Age-standardized estimates in panel B adjust for the changing age structure of the population. If the U.S. population structure remained unchanged at the 2010 level, prevalence of any pain would increase by only 8%. The relative increases would also be smaller for joint and low back pain (14% and 13%, respectively) but larger for facial/jaw pain (15%) and especially headache/migraine (10%), which occurs more frequently at younger ages.

Table 2 shows the characteristics of the target population in 2002 and in 2018, as well as the *p* values of tests for differences between these two years. Overall, there were noticeable (and statistically significant) changes in most characteristics. The population in 2018 was older and more non-White, with a larger proportion of foreign-born and college graduates compared with 2002. Health behaviors changed as well: smoking declined sharply, and the proportion meeting federal guidelines for physical activity increased, but so did excessive alcohol use and BMI. The prevalence of chronic conditions generally increased except for the prevalence of respiratory conditions, which declined.

Figure 1 visualizes the age-adjusted trend in “any pain” by age group and sex, summarizing results from our analytic Step 2. Overall, in all age/sex groups, pain increased monotonically. For men and women aged 45–64, there was stagnation or even a decrease (not significant) in the last few years, while for those aged 65–84, the increase appears to accelerate. Over the full 17 years, however, the trend is roughly linear for the three age groups. The linearity is substantively problematic because it indicates continued increases in pain, but convenient methodologically, as it allows us to employ a parsimonious linear specification for the trend in subsequent analyses. Additional figures showing trends by income are in the online appendix.

Table 3 summarizes findings from analytic Step 3 to examine pain trends across population subgroups, using demographics-adjusted logistic models of each pain indicator. As noted earlier, the time trend is scaled to a 0–1 range so that the odds ratio for pain trend shown in the table can be interpreted as the relative change in pain prevalence over the 17-year observation period. Several findings are important. First, net of changes in demographic composition, U.S. adults had 24% higher odds of reporting pain in 2018 compared with 2002. Second, the prevalence of pain in all individual pain sites significantly increased, especially joint pain (26% higher odds in 2018 compared with 2002) and low back pain (20% higher odds). Third, the pain increases were systemic: almost all groups experienced a significant increase pain over time. Fourth, across the 204 separate models summarized in Table 3, plus additional ones estimated as robustness checks, no group and no pain indicator showed a significant decrease. Fifth, at the same time, the pain trends varied significantly across groups. Male, Black, lower-income, and less-educated respondents experienced significantly steeper increases in at least some pain sites compared with their female, White, higher-income, and higher-educated counterparts. The trends were also steeper for older adults and those from earlier generations.^2^

Tables 4–6 present findings from analytic Step 5 (investigation of correlates of pain trends). Table 4 summarizes how each covariate individually changes the pain trend. The covariates are ordered from “their inclusion attenuated the pain trend the most” to “their inclusion increased the pain trend the most.” Among adults aged 25–44 and 45–64, the coefficients whose inclusion attenuated the pain trend the most are psychological distress (K6), alcohol use, and BMI; controlling for smoking, physical activity, and educational attainment resulted in a steeper pain trend gradient, making these suppressor covariates. Among adults 65–84, controlling for BMI, hypertension,^3^ and diabetes attenuated the pain trend the most; education, income, and physical activity yielded a steeper pain trend.

Table 5 shows how all covariates jointly correlate with pain. Net of all included covariates, the odds ratio for the pain trend estimated for the total population is 1.11 (*p* < .001), compared with 1.24 in Table 3. We also see that age group differences become more pronounced: for adults aged 65–84, the upward pain trend remains largely unchanged from the demographics-adjusted models in Table 3 (OR = 1.30, *p* < .001 vs. OR = 1.35, *p* < .001). In contrast, the pain trend in the 25–44 age group becomes flat (OR = 1.02, nonsignificant, vs. OR = 1.17, *p* < .001). Another key take-away is that the effect of covariates on pain level is in the direction expected based on prior literature. Being female, White, having lower income, smoking, excessive alcohol use, and high BMI are all correlated with higher odds of reporting pain, as are most chronic conditions.

Table 6 shows findings from the nonlinear decomposition in which we decomposed the difference in pain between “early” in the observation period (2002–2004) and “late” (2016–2018) to differences in composition and differences in covariate effects. In the total sample, pain increased by 5.9 percentage points. About 67% of this increase can be attributed to differences in population composition, and the remaining 33% either is due to changes in the effects of covariates or is unexplained. This aggregate decomposition differs across age. Among adults aged 25–44, 71% of the pain difference is due to changes in population composition. The respective percentages are 51% among the middle-aged and 28% among older adults. These findings fit well with results in the prior analytic step shown in Table 5: most of the pain increase over time for young adults could be explained by differences in population characteristics, whereas most of the pain increase among older adults was not explained.

**Fig. 2:**
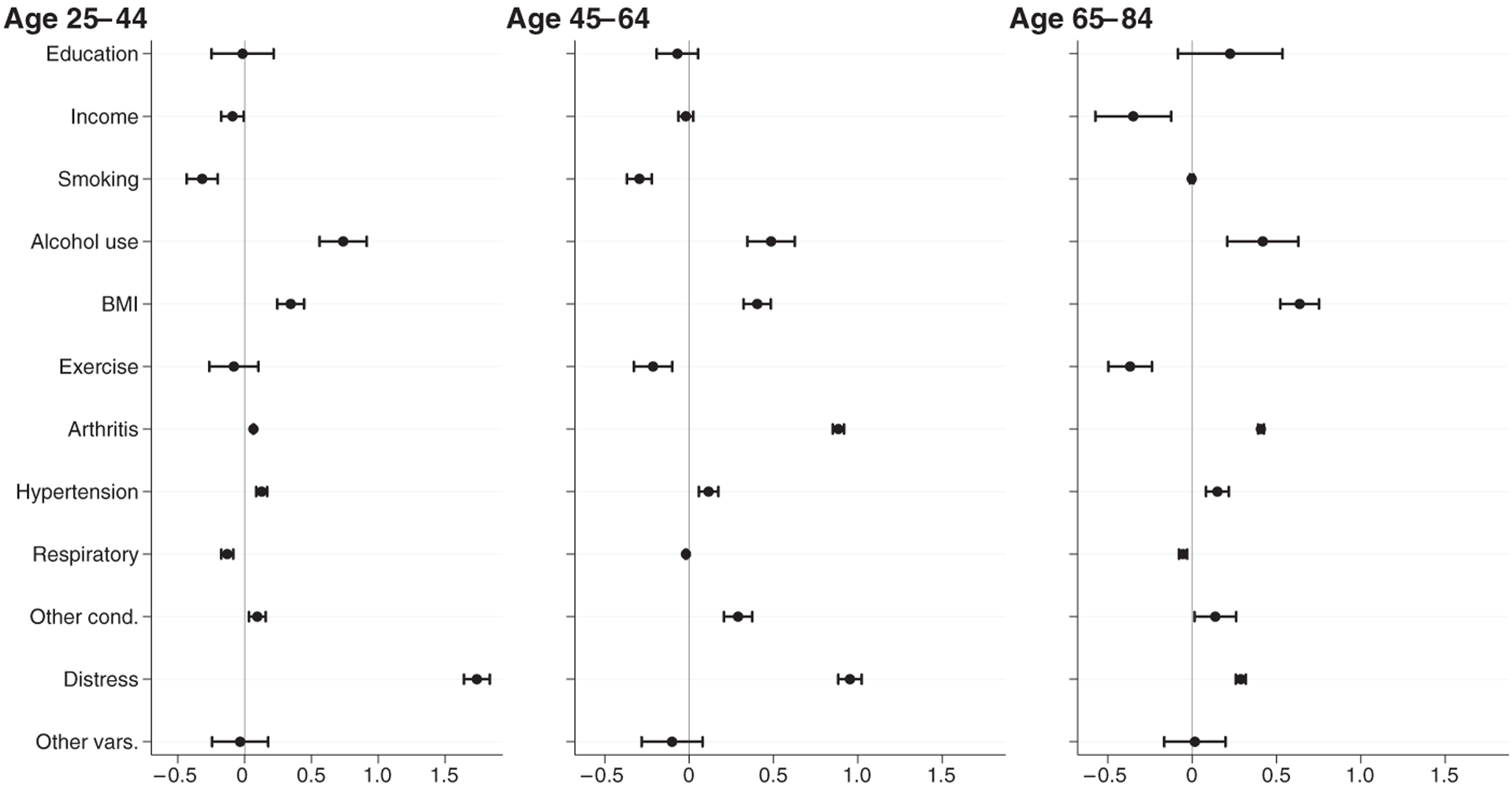
Contribution of changes in composition, by age group. The figure shows coefficients and their 95% confidence intervals (CIs) estimating the contribution of compositional differences between 2002–2004 and 2016–2018 populations to pain prevalence differences. For several estimates, the standard errors are small enough that the plotted CI is not clearly visible around the point estimate.

The detailed decomposition for the total population shows that changes in the composition (panel B1) of most characteristics were significantly related to the pain increase. Among adults aged 25–44, psychological distress is the most important covariate: about half of the pain increase is linked to the increase in distress. Changes in alcohol use, BMI, and smoking are also highly salient. Similarly, in the 45–64 age group, changes in psychological distress are strongly linked to pain increases, as are alcohol use and BMI. However, in this age group, arthritis is the most salient correlate: 20% of pain increase in this group is due to an increase in arthritis. Among older adults, the most important covariate is BMI; and alcohol use and arthritis are also important, but to a lesser degree. These covariates are fairly similar to those that were prominent for pain trends individually (Table 4), where psychological distress, alcohol use, and BMI attenuated the pain trended the most. Figure 2, which visualizes the estimated coefficients, highlights that changes in the population composition with respect to psychological distress, alcohol use, and BMI were significant predictors of changes in pain prevalence in all three age groups and had large effect sizes. The coefficient for arthritis is particularly salient in the 45–64 age group. It is also sizable among adults aged 65–84, for whom hypertension and other chronic conditions also have significant if modest effect sizes.

The estimation of changes in coefficients (Table 6, panel B2) yielded large standard errors, so only a few covariates were statistically significant. The model indicates that changes in the relationship between arthritis and pain in older adults, between physical activity and pain in adults aged 25–44 and 45–64, and between alcohol use and pain in younger adults all contributed significantly to the pain increase. All these significant effects were also picked up as significant interactions in the fully adjusted interaction models, as indicated by the directional (+ or −) sign and associated *p* value in Table 5.

## Discussion

Chronic pain is a common, disabling, and both personally and economically costly health problem. Assessing trends in its prevalence and social distribution is crucial for understanding and ultimately improving U.S. population health. In this study, we analyzed pain trends from 2002 to 2018 in the U.S. adult population (ages 25–84), tested group differences in the trends, and identified socioeconomic, behavioral, psychological, and medical factors correlated with the trends.

Already in 2002, pain was very common, affecting 49% of American adults. By 2018, prevalence had risen to 54%—an increase of approximately 10% in relative terms, corresponding to an extra 10.5 million Americans experiencing pain.^4^ Moreover, these increases were systemic: most population groups experienced increasing pain prevalence in most pain sites. Indeed, we found no population group and no pain site for which pain declined significantly. The sites with the steepest relative increases (joint and low back pain) were also those with the highest prevalence at baseline; correspondingly, the summary “any pain” measure increased substantially as well. This upward trend corroborates prior reports of pain increases among U.S. Whites aged 45–54 (Case and Deaton 2015) and adults older than 50 (Grol-Prokopczyk 2017; Zimmer and Zajacova 2020), as well as a rise in painful health conditions in the total U.S. population (Nahin et al. 2019).

Although all groups experienced increasing pain over time, we found important differences that suggest attenuation of demographic (sex and Black White) disparities but amplification of socioeconomic disparities. We replicated prior findings that men and racial/ethnic minorities are less likely to report pain than women and Whites, respectively (Bartley and Fillingim 2013; Kennedy et al. 2014; Nahin 2015). (These static comparisons are evident in Table 5 as significant positive effects for females and negative effects for racial/ethnic minorities.) Men and Black adults, however, experienced significantly steeper increases in “any pain” and some specific pain sites (bolded coefficients in Table 3), leading to reductions in disparities across these demographic groups. We underscore that these disparity reductions should not be celebrated: rather than less healthy groups faring better over time, here is a case of all groups—especially previously better-off ones—faring progressively worse.

Also worrisome are the significant and growing chronic pain disparities by SES (Table 3). From 2002 to 2018, adults whose family income was at least four times the poverty level experienced a 14% increase in the odds of pain, whereas adults with less than twice the poverty level—corresponding to a 2018 family income below $50,000 for a family of four—experienced roughly a 40% increase. Educational disparities mirrored those by income: college graduates experienced a 17% increase in the odds of pain, whereas adults who never attended college experienced a 40% increase. These findings are consistent with other studies that found increasing socioeconomic disparities in other health outcomes, including other chronic conditions, disability, and mortality (Sasson 2016; Singh and Jemal 2017; Zajacova and Montez 2017b).

As anticipated, the picture of pain trend correlates is complex for several reasons. First, correlates of pain *trends* are not necessarily the same as correlates of pain *levels.* Our study focused on the former, exploring how changes in the distribution of covariates in the population over time, and/or changes in the effects of these covariates, correlate with changes in pain. Second, pain is influenced by a complex web of causes, from sociodemographic characteristics to intermediate factors like health behaviors to proximal factors like pain-producing health conditions (Craig and Fashler 2013). Moreover, as highlighted in the WHO social determinants of health framework (Solar and Irwin 2010), all individual-level determinants and their effects on health are inextricably grounded in a given socioeconomic-political context, encompassing a broad array of upstream institutional and cultural influences. Although data limitations prevented us from exploring the distal contextual factors, the ubiquitous pain increases we describe here suggest that broad changes in the socioeconomic-political context may underlie these undesirable trends. A third reason why this picture is complex is that even though we restricted our attention to individual level variables, there are likely recursive causal effects. For example, low income may raise the risk of pain via mechanisms such as stress and depression or poor health behaviors; at the same time, chronic pain may increase stress and depression or impact health behaviors. Although our cross-sectional data do not permit us to unpack such causal effects, we highlight important correlations that we hope will be explored further in future research.

Psychological distress, a widely used index combining depressive and anxiety-related symptoms (Kessler et al. 2002; Kessler et al. 2003), was the most prominent correlate of pain increases in adults under 65. In the 25–44 age group, for example, the increase in psychological distress accounted for 50% of the difference in pain prevalence between the start and end of the study period (Table 6). The prominent role of distress should not be surprising. Psychological distress and depression are widely considered risk factors for chronic pain (Gatchel et al. 2007; Wilson et al. 2019), although the associations are clearly bidirectional (Janevic et al. 2017; van Hecke et al. 2013). We also note intriguing parallels with the literature on trends in disability and mortality, which has pinpointed despair as a potential critical factor in their worrisome increases (Case and Deaton 2015; Monnat and Brown 2017). Perhaps in addition to “deaths of despair,” we need to understand and address “pain of despair.”

Health behaviors represent the second set of salient characteristics associated with pain trends, again especially among adults younger than 65. From 2002 to 2018, average body weight increased significantly in the United States, as did excessive alcohol use (Table 2). At the same time, the percentage of current smokers declined from 22% to 15%, and physical activity increased. All these lifestyle variables predicted pain trends both independently and jointly. For example, among adults aged 25–44, 21% of the pain increase was attributable to changes in alcohol use, and an additional 10% was attributable to increased BMI (Table 6). This result coincides with a recent report on older U.S. adults, for whom 10% to 32% of the pain increase from 1992 to 2016 was attributable to the increases in BMI during that time (Stokes et al. 2020). The associations between health behaviors and pain trends described here mirror those for health behaviors and pain *prevalence* (Gale et al. 2012; Katz 2006; Okifuji and Hare 2015). There may also be a vicious spiral among psychological stress, distress, “self-destructive health behaviors” (Stein et al. 2017:1541), and pain: alcohol use and obesity may reflect maladaptive coping mechanisms for social stress (Lazarus and Folkman 1984; Park and Iacocca 2014), culminating in an increased risk of pain.

A complex pattern pertains to income and education. Although pain trends differed significantly between adults with high versus low income and education, and education was a prominent suppressor of the pain trend on its own, these characteristics became largely nonsignificant in the decomposition analysis (Tables 4–6). We surmise that the decomposition analysis—which included intermediate and proximate correlates of pain, such as health behaviors and chronic conditions—effectively “explained” the links between changing distributions of socioeconomic factors and pain trends (Brunello et al. 2016; Link and Phelan 1995). However, more research is needed to understand how social factors and pain changes over time are connected.

We conducted all analyses separately by age group because of anticipated differences across different life course stages. Indeed, although pain increased in all age groups, we found important differences. First, older adults experienced steeper pain increases than younger adults in most pain sites (Table 3). Second, the correlates of the pain trends differed by age. For younger and middle aged but not older adults, for instance, psychological distress was the most prominent correlate of trends (Tables 4 and 6; Figure 2). Alcohol use was a more prominent correlate in the youngest group, whereas arthritis and other conditions had greater importance in the older groups. Finally, a higher proportion of the pain trend remained unexplained in older versus younger ages. The pain trend was steeper for older adults net of only demographics (Table 3) and in fully adjusted models (Table 5) compared with their younger counterparts; in decomposition analyses a larger percentage of pain increase for older age groups was attributed to different effects of correlates or remained unexplained (Table 6, panel A). We describe these tendencies in terms of age groups; however, whether these differences in fact reflect age, birth cohort, and/or period influences is difficult to disentangle (Bell and Jones 2014, 2018). However, there clearly are age or generational differences in pain trends, and we hope that future studies, ideally with longitudinal data, might gain traction on this issue.

We note three limitations of the NHIS data. First, their cross-sectional structure is a limitation because it restricts our analyses to correlational associations. The decomposition offers a counterfactual perspective that slightly enhances our ability to understand the complex links between pain and its covariates. It is also useful to remember that our focus is on linking changes in the population distribution of covariates to changes in pain rather than on identifying causes of pain in individuals. Ultimately, however, we cannot overcome the potential endogeneity in our models, and thus we urge caution in interpreting the findings. A second limitation, which does not impact our findings about trends but does complicate cross study comparisons, pertains to the specific questions used to assess pain. The NHIS asks about only five sites of pain and excludes others, including highly distressing ones like abdominal pain (Townsend et al. 2005). In a 2010 supplement, the NHIS included “persistent pain” questions, defined as frequent or constant pain during the past three months. Under this definition, 19% of U.S. adults (age 18+) reported pain (Kennedy et al. 2014). We thus urge caution in comparing pain prevalence across data sets with different definitions and operationalizations. Finally, in the NHIS data, questions about pain frequency, severity, and pain’s impact on everyday functioning are either not available or available only for a subset of years or respondents. Future studies should explore other data sources with such information to gain a fuller picture of pain burden in the United States.

A critical question about our findings is whether pain prevalence is *really* increasing or whether our findings are artifacts of changing reporting styles. That is, are Americans experiencing more pain or simply reporting more pain? There is no objective biomarker for pain, so researchers and clinicians rely on self-reports (Unruh et al. 2013:1), and social context shapes how pain is perceived, experienced, and reported (Craig and Fashler 2013). Indeed, a number of institutional and cultural developments in the United States could have potentially encouraged greater pain reporting since the mid-1990s. The influential “pain as the fifth vital sign” campaign launched in 1995, resulting in more aggressive assessment and treatment of pain (Scher et al. 2018). Simultaneously, pharmaceutical companies developed and aggressively marketed numerous new and reformulated opioid analgesics, most notoriously Purdue Pharma’s OxyContin, introduced in 1996 (Jones et al. 2018; Tompkins et al. 2017). Americans may have begun reporting pain more readily, in the (mistaken) belief that chronic pain was effectively and safely treatable. It is theoretically possible that such reporting changes would manifest not only in clinical settings but also in surveys such as the NHIS.

On the other hand, several forms of evidence argue against the idea that the rise in U.S. pain prevalence is artifactual. First, Nahin et al.’s (2019) documentation of steep rises in U.S. pain from 1997 to 2014 relies on diagnosed pain-related health conditions and ICD-9 categories (e.g., osteoarthritis, temporomandibular joint disorder), which are arguably more resistant to changing reporting norms than questions about less well-specified pain. Next, studies provide evidence that U.S. pain prevalence increased *before* the regulatory and commercial developments of the mid-1990s/early 2000s (Zimmer and Zajacova 2020) as well as *after* the CDC’s 2011 declaration of an opioid epidemic and the subsequent backlash against the “fifth vital sign” and opioid manufacturers (Jones et al. 2018). That is, pain levels increased before, during, and after the cultural shifts theorized to shape reporting. Furthermore, pain appears to be increasing globally (Shupler et al. 2019; Zimmer et al. 2020), including in Western European countries with different therapeutic regimes and much tighter regulation of pharmaceutical marketing than found in the United States (Meyer et al. 2020). The findings we report here are thus not unique to the U.S. political-economic context. Additionally, two recent analyses (using data from 2004–2016 and 2002–2010) found no change over time in the association between self-reported pain and more objective measures of pain-related function—specifically, walking speed and work disability (Grol-Prokopczyk et al. 2019; Wynne-Jones et al. 2018). Finally, our own findings show that the correlation between most health conditions and pain has not changed over time (Table 6, panel B2). However, we also acknowledge that among older adults, a large component of the pain increase could not be explained by the changing distribution of pain determinants (Table 6, panel A), leaving open the possibility of reporting changes as partial contributors to the observed trends. Overall, however, although reporting factors may be at play and should be explored further, evidence of rising pain prevalence now comes from too many countries, contexts, and data sources to be easily dismissed.

There are highly plausible potential mechanisms for rising U.S. pain prevalence, including some that were assessed in our study. For instance, rising obesity may contribute to the increase in pain prevalence (Stokes et al. 2020). Obesity can cause or exacerbate pain via multiple mechanisms, such as mechanically in terms of stress on the musculoskeletal system (McVinnie 2013) or chemically via inflammatory cytokines (Okifuji and Hare 2015). One potential mechanism that we were unable to assess is the rise in use of prescription opioid analgesics. Disturbingly, there is no evidence that long-term use of opioid “painkillers” is effective in treating chronic pain (Chou et al. 2015; Kissin 2013; Sommer et al. 2020). A recent randomized yearlong trial actually found that opioids reduced pain *less* than nonopioids like Tylenol (Krebs et al. 2018), and other studies have found that prescription opioids predict more intense pain, lower functioning, higher disability, and higher healthcare utilization among chronic pain patients (Eriksen et al. 2006; Morasco et al. 2017). Of particular relevance to the current study is the growing evidence from both human and animal-model studies that opioid use can exacerbate pain in the long term and thus may contribute to its increasing prevalence in the population (Ballantyne and Shin 2008; Feehan and Zadina 2019; Green-Fulgham et al. 2019; Lee et al. 2011). In summary, our findings in this study are not an argument for increased opioid use; in contrast, we posit that opioids may have contributed to the rise of pain prevalence in the United States.

## Conclusion

This study has documented steep, sustained, and pervasive increases in chronic pain among Americans across the adult life span. This is a concerning finding that should stimulate new research in demography and other social sciences. We found that key correlates of the rise in pain prevalence include not only specific diagnoses, such as arthritis, but also psychological distress, increased body weight, and heavier alcohol use—factors that highlight the psychosocial roots of pain in populations (Carr 2016). Given its links to both physical and psychological well-being, chronic pain could be conceptualized as a holistic measure of population health and could supplement the disability and longevity measures that have long been the central focus of health demography. Our findings support the need for broad interdisciplinary research on, and interventions for effective responses to, the growing problem of pain in the United States.

## Supplementary Material

Online Appendix

## Figures and Tables

**Table 1:** Pain prevalence and age-standardized pain prevalence among U.S. adults aged 25–84, 2002 and 2018

	Any Pain	Joint	Back	Neck	Headache/Migraine	Facial/Jaw
A. Prevalence						
2002	49.1	26.9	27.4	14.8	15.0	4.7
2018	53.8	32.5	31.4	17.2	15.6	5.3
Percentage change	9.7	20.6	14.9	15.9	4.5	12.6
Percentage point change	4.7	5.5	4.1	2.4	0.7	0.6
Test of difference ( *p* value)	<.001	<.001	<.001	<.001	.097	.020
B. Age-Standardized Prevalence						
2002	49.4	27.7	27.5	14.9	14.6	4.7
2018	53.2	31.5	31.1	17.1	16.1	5.4
Percentage change	7.8	13.9	12.9	15.4	9.8	14.6
Percentage point change	3.9	3.8	3.5	2.3	1.4	0.7
Test of difference ( *p* value)	<.001	<.001	<.001	<.001	.106	.023

*Notes:* Panel A shows weighted proportions in the total sample. Panel B shows weighted age-standardized proportions using the 2010 U.S. population age structure. Prevalence and age-adjusted prevalence for each single year from 2002 to 2018 are available in the online appendix.

*Source:* NHIS 2002 and 2018.

**Table 2:** Characteristics of the target population, U.S. adults aged 25–84, 2002 and 2018, and difference across the two years

	2002	2018	Difference (*p*)
Demographics			
Age, mean (SD)	48.2 (15.0)	50.2 (15.6)	<.001
Female	52.1	51.8	.584
Race			<.001
White	74.1	64.6	
Black	11.1	12.1	
Hispanic	10.5	15.7	
Other	4.4	7.6	
Proxy responder	1.0	1.5	<.001
Foreign-born	14.1	19.4	<.001
Interview not English	4.9	5.9	.017
Region			<.001
Northeast	19.4	17.7	
Midwest	24.2	21.5	
South	37.0	37.4	
West	19.5	23.4	
Social Ties			
Not married	35.0	40.9	<.001
Children at home	45.7	42.6	<.001
SES			
Education			<.001
Less than high school	19.0	13.8	
High school	26.5	21.0	
Some college	27.5	28.8	
Bachelor’s degree or more	27.0	36.4	
Not employed (currently)	34.1	35.2	.074
Work status prior year			<.001
Worked all 12 months	58.7	57.3	
Worked 1–11 months	12.3	11.3	
Did not work for pay	29.0	31.4	
Income			<.001
Below poverty level	9.7	9.4	
1–1.9 times poverty level	16.2	16.4	
2–3.9 times poverty level	32.0	28.1	
4 times poverty level	42.1	46.2	
Rents (not a homeowner)	25.2	30.8	<.001
Health Behaviors			
Smoking			<.001
Never	53.3	61.2	
Former	24.8	24.0	
Current	22.0	14.8	
Alcohol use			<.001
Never	20.4	16.8	
Former	16.4	15.0	
Current moderate	43.9	42.1	
Current excessive	19.3	26.2	
BMI, mean (SD)	27.2 (5.6)	28.5 (6.4)	<.001
Physical activity (meets guidelines)	41.1	51.1	<.001
Chronic Conditions			
Arthritis	23.0	25.9	<.001
Cancer	7.7	10.0	<.001
Respiratory disease	5.8	5.0	<.001
Heart disease	11.8	12.5	.037
Diabetes	8.3	13.9	<.001
Hypertension	26.8	34.2	<.001
Kidney disease	1.4	2.6	<.001
Liver disease	1.3	2.0	<.001
Stroke	2.5	3.2	<.001
Distress (K6), mean (SD)	2.2 (3.8)	2.8 (4.1)	<.001

*Notes:* Adjusted for the complex survey design. Difference between 2002 and 2018 in categorical variables is tested with a design-based *F* test (equivalent to a chi-squared test but appropriate for complex survey data); for continuous variables we test the equality of survey design-adjusted year-specific means.

*Source:* NHIS 2002 and 2018.

**Table 3:** Pain trends in aggregate population and in subgroups, logistic models, 2002–2018

	Any Pain	Joint	Back	Neck	Headache/Migraine	Facial/Jaw
Full Sample	1.24***	1.26***	1.20***	1.17***	1.14***	1.13***
By Age Group						
25–44	1.17***	1.24***	1.13***	1.11**	1.15***	1.15**
45–64	1.26***	1.24***	1.22***	1.21***	1.19***	1.12*
65–84	1.35***	1.29***	1.31***	1.27***	1.17**	1.17*
By Cohort (generation)						
Greatest Generation (1918–1932)	**1.08**	**1.18**	1.26*	**0.97**	**0.97**	1.22
Silent (1933–1945)	1.54***	1.47***	1.36***	1.33***	**1.08**	1.17
Early Boomer (1946–1954)	1.28***	1.34***	1.22**	1.20*	1.23*	1.23
Late Boomer (1955–1964)	1.19**	1.14*	1.22***	1.16*	**1.02**	1.11
Generation X (1965–1980)	1.19***	1.30***	1.11**	1.12*	1.17***	1.05
Millennial (1981–1993)	1.21*	1.32*	1.13	1.02	1.22*	0.98
By Sex						
Men	1.28***	1.31***	1.24***	1.24***	1.23***	1.18**
Women	1.19***	1.22***	1.16***	1.12***	1.10***	1.11**
By Race						
White	1.23***	1.27***	1.18***	1.17***	1.16***	1.13**
Black	1.37***	1.23***	1.39***	1.24***	1.15*	1.12
Hispanic	1.20***	1.20***	1.15**	1.10	1.06	1.24*
Other	1.11	1.27**	1.04	1.16	1.01	0.99
By Region						
Northwest	1.15***	1.27***	1.05	1.03	1.10	1.22*
Midwest	1.17***	1.20***	1.12**	1.16***	1.25***	1.11
South	1.29***	1.26***	1.33***	1.26***	1.11**	1.03
West	1.30***	1.33***	1.19***	1.17***	1.13**	1.27***
By Interview Type						
Self-respondent	1.23***	1.26***	1.19***	1.16***	1.14***	1.13***
Proxy	1.37*	1.30	1.27	1.80***	1.67**	1.60
By Nativity						
U.S.-born	1.25***	1.26***	1.21***	1.18***	1.16***	1.12**
Foreign born	1.15***	1.26***	1.10*	1.08	1.00	1.23*
By Language of Interview						
English	1.24***	1.26***	1.21***	1.17***	1.14***	1.13***
Not English	1.14*	1.25**	**0.97**	1.06	1.05	1.17
By Education						
Less than high school	1.40***	1.43***	1.41***	1.34***	1.24***	1.31***
High school	1.40***	1.43***	1.40***	1.30***	1.20***	1.08
Some college	1.29***	1.30***	1.25***	1.13***	1.24***	1.08
Bachelor’s degree or more	1.17***	1.18***	1.15***	1.19***	1.17***	1.24***
By Income						
Below poverty level	1.38***	1.45***	1.39***	1.34***	1.15**	1.19*
1–1.9 times poverty level	1.42***	1.35***	1.38***	1.37***	1.30***	1.25**
2–3.9 times poverty level	1.29***	1.35***	1.24***	1.18***	1.16***	1.19**
4 times poverty level	1.14***	1.16***	1.09***	1.08*	1.08*	1.06

*Notes: N* = 441,707 in aggregate population. Each cell shows the odds ratio for the effect of time. We estimated logistic models of “any pain” as a function of a continuous linear time trend plus basic controls. The time trend is scaled to range from 0 to 1 so that the odds ratio can be interpreted as the difference in the odds of reporting pain at the end of the observation period relative to the beginning (2018 vs. 2002). The controls are age, sex, race, region, foreign-born, language of interview, and proxy respondent status. Models that stratify for a given characteristic omit that characteristic from the list of covariates except for age, which is included in the age stratified and cohort stratified analyses. Missing values are imputed as discussed in the Methods section; estimation takes into account NHIS complex sampling design. The bolded cells indicate statistically significantly different trends across groups: we estimated additional logistic models of each pain measure for which we did not stratify but interacted the variable of interest (such as sex, race, and cohort) with the linear continuous time trend. For parsimony, rather than show a full set of results from these interaction models, we highlight in this table those characteristics that interacted significantly (*p* < .05) with the time trend. The omitted categories in these models were, respectively, age 25–44, Millennial cohort, male, White, Northwest, self-respondent, U.S.-born, language of interview was English, and the highest educational and income categories. Thus, for instance, the bolded coefficients for the Greatest Generation indicate that the “any pain,” joint, neck, and headache/migraine pain trend for this generation differs significantly from the pain trend in the Millennial generation, net of included covariates.

*Source:* NHIS 2002–2018.

* *p* < .05;

** *p* < .01;

*** *p* < .001

**Table 4:** Percentage change in pain trend when adjusting for single covariates in models of pain trends, 2002–2018

Age 25–44		Age 45–64		Age 65–84		Total	
Covariate	% Change	Covariate	% Change	Covariate	% Change	Covariate	% Change
Distress (K6)	−76	Distress (K6)	−36	BMI	−21	Distress (K6)	−40
Alcohol Use	−34	BMI	−23	Hypertension	−13	BMI	−25
BMI	−31	Alcohol Use	−14	Diabetes	−9	Alcohol Use	−20
Hypertension	−16	Diabetes	−9	Kidney Cond.	−7	Hypertension	−13
Homeowner	−14	Homeowner	−8	Alcohol Use	−6	Diabetes	−9
Diabetes	−8	Hypertension	−7	Cancer	−6	Homeowner	−8
Married	−7	Married	−4	Distress (K6)	−5	Cancer	−3
Liver Cond.	−3	Cancer	−3	Liver Cond.	−2	Married	−3
Stroke	−3	Liver Cond.	−1	Homeowner	−1	Liver Cond.	−2
Heart Cond.	−3	Stroke	−1	Married	0	Kidney Cond.	−2
Cancer	−1	Kidney Cond.	−1	Children	0	Stroke	0
Arthritis	0	Income	0	Stroke	1	Children	0
Employment	1	Children	1	Smoking	2	Arthritis	1
Kidney Cond.	1	Respiratory	6	Arthritis	4	Employment	1
Prior Empl.	1	Prior Empl.	7	Employment	4	Prior Empl.	2
Children	2	Employment	7	Prior Empl.	4	Heart Cond.	4
Income	3	Heart Cond.	8	Respiratory	5	Income	6
Phys. Activity	9	Arthritis	14	Heart Cond.	7	Respiratory	7
Respiratory	10	Education	17	Phys. Activity	12	Phys. Activity	14
Smoking	28	Phys. Activity	18	Education	14	Smoking	19
Education	30	Smoking	24	Income	14	Education	20

*Notes:* Each cell in the table shows the percentage change in the log odds of the coefficient for time trend when each covariate is added to a logistic model of “any pain” estimated as a function of demographics (age, sex, race, region, proxy respondent status, nativity, and language of interview). In each age group, the individual covariates are then arranged in order from the most attenuated to the most strengthened coefficient associated with the time trend. Variables with negative % values could be understood as “mediators,” and those with positive % values could be thought of as “suppressors.”

*Source:* NHIS 2002–2018.

**Table 5:** Logistic models of any pain, adjusted for all covariates, 2002–2018

	Age 25–44		Age 45–64		Age 65–84		Total	
Time Trend	1.02		1.19***		1.30***		1.11***	
Age	1.01***		0.99***		0.99**		1.00***	+**
Female	1.35***		1.27***		1.29***		1.32***	−*
Race (ref. = non-Hispanic White)								
Black	0.72***	+***	0.77***		0.83***		0.76***	+**
Hispanic	0.86***		0.84***		0.90*		0.85***	
Other	0.77***		0.83***		0.86**		0.80***	
Region (ref. = Northeast)								
Midwest	1.02		1.03		1.16***		1.05**	
South	0.99		1.03		1.18***		1.04*	
West	1.15***		1.19***		1.26***		1.19***	+*
Proxy Responder	0.78*		0.75***		0.78***		0.75***	
Foreign-born	0.87***		0.84***		0.89**		0.86***	
Interview Not in English	0.83***		0.97		0.94		0.89***	
Not Married	0.92***		0.94***		0.89***		0.91***	
Children at Home	1.11***		1.05**		1.01		1.11***	
Education (ref. = bachelor’s degree+)								
Less than high school	1.04		1.01		1.01		1.03	
High school	1.01		0.98		0.94*		0.99	
Some college	1.18***		1.10***		1.05		1.13***	
Not Employed (currently)	1.03		1.05		1.08		1.02	
Worked Last Year (ref. = 12 months)								
Worked 1–11 months	1.09***	−**	1.14***		1.05		1.10***	−***
Did not work	0.94*		1.12***		0.98		0.97	
Income (ref. = 4 times poverty level)								
Below poverty level	1.12***		1.13***		1.15**		1.13***	
1–1.9 times poverty level	1.12***		1.16***		1.08*		1.11***	
2–3.9 times poverty level	1.06**		1.05*		1.05		1.04**	
Rents (not a homeowner)	1.01		0.96*		1.02	+*	0.98	+*
Smoking (ref. = never)								
Former	1.26***		1.16***		1.11***		1.17***	
Current	1.36***	+**	1.20***	+*	1.15***		1.30***	+**
BMI	1.02***	−**	1.02***		1.03***		1.02***	
Alcohol Use (ref. = never)								
Former	1.43***	−**	1.41***		1.13***		1.35***	
Current moderate	1.46***		1.42***		1.19***		1.39***	
Current excessive	1.63***		1.53***		1.18***		1.52***	
Physical Activity	0.99	+*	1.06**	+***	1.13***	+**	1.03**	+***
Chronic Conditions								
Arthritis	5.93***		6.33***		6.01***	−***	6.09***	−**
Cancer	1.36***		1.19***		1.12***		1.14***	
Diabetes	1.06		0.99	+**	1.05	+*	1.04*	+***
Hypertension	1.38***		1.15***		1.17***		1.22***	
Kidney disease	1.54***		1.39***		1.42***		1.44***	
Liver disease	1.38***		1.74***		1.49***		1.64***	
Stroke	1.65***		1.13*		1.07		1.11***	
Respiratory disease	1.97***		1.74***		1.33***	+*	1.63***	
Heart disease	1.82***		1.41***		1.25***		1.37***	
Distress (K6)	1.17***		1.15***		1.13***		1.16***	−*

*Notes: N* = 441,707. Multiply imputed models; estimation takes into account NHIS complex sampling design. The odds ratios and associated *p* values shown are from an additive fully adjusted model of “any pain.” The column with a directional sign (+ or −) and *p* value indicates significant interactions with time. We estimated additional fully adjusted logistic models of pain in which we interacted all covariates with the linear continuous time trend. For parsimony, we do not show the full results from these models, but we indicate which covariates had a significant interaction with time and in which direction. For instance, among adults 25–44, the coefficient for Black has a + sign and *p* < .001. That indicates that the pain trend for Blacks is significantly different—steeper—than the trend for Whites in the fully adjusted and fully interacted models.

*Source:* NHIS 2002–2018.

* *p* < .05;

** *p* < .01;

*** *p* < .001

**Table 6:** Nonlinear decomposition of pain prevalence differences in 2002–2004 and 2016–2018 into changes in composition versus changes in coefficients

	Age 25–44	Age 45–64	Age 65–84	Total
A. Total Decomposition				
Pain prevalence 2002–2004	43.7***	54.4***	57.6***	50.2***
Pain prevalence 2016–2018	47.1***	59.2***	63.0***	56.1***
Difference (percentage point)	3.5***	4.7***	5.4***	5.9***
Decomposed to:				
Composition	2.4***	2.4***	1.5***	3.9***
Coefficient	1.0*	2.3***	3.9***	1.9***
Expressed in percentage				
% due to composition difference	70.5	51.2	28.2	66.6
% due to coefficient difference	29.4	48.8	71.8	33.4
B. Detailed Decomposition				
B1. Percentage due to compositional changes in:				
Education	−0.5	−1.5	4.2	−0.2
Income	−2.6	−0.4	−6.5**	−1.8***
Smoking	−9.2**	−6.3***	0.0	−4.1***
Alcohol use	21.3***	10.3***	7.8***	9.0***
BMI	10.0***	8.6***	11.9***	8.1***
Physical activity	−2.3	−4.5***	−6.8***	−3.5***
Arthritis	1.9***	18.8***	7.6***	30.3***
Hypertension	3.7***	2.4***	2.8***	4.8***
Respiratory	−3.8***	−0.4***	−1.0***	−0.2***
Other conditions	2.7**	6.2***	2.6*	7.2***
Distress (K6)	50.2***	20.2***	5.4***	17.1***
Other control variables	−1.0	−2.2	0.3	0.1
B2. Percentage due to coefficient changes in:				
Education	−2.1	−1.1	1.3	−0.6
Income	−1.3	−7.7	−3.6	−3.2
Smoking	−8.3	−5.5	2.7	−3.0
Alcohol use	19.3**	2.3	−5.4	2.5
BMI	−68.6	−3.7	41.7	−10.5
Physical activity	32.4*	33.9**	22.6	27.5***
Arthritis	−3.4	−13.4	−32.0**	−10.6**
Hypertension	−5.5	0.3	−3.6	−2.7
Respiratory	−0.5	−1.5	4.1	−0.5
Other conditions	−8.6*	10.9	2.4	1.0
Distress (K6)	−1.1	−8.9	−3.6	−6.1
Other control variables	−68.2	46.7	88.3	−2.4
*N*	62,018	57,573	35,971	155,562

*Source:* NHIS 2002–2004 and 2016–2018.

* *p* < .05;

** *p* < .01;

*** *p* < .001

## Footnotes

1 Our analysis focuses on chronic pain (which we often abbreviate as “pain”). Chronic pain is typically Conceptualized as pain that “lasts more than several months” (Institute of Medicine. 2011:33). For instance, the ICD-11 defined chronic pain as “persistent or recurrent pain lasting Longer than 3 months” (Treede et al. 2015:1004). The question Wording in the National Health Interview Survey, described in the Methods section, is within the spectrum of commonly used definitions of chronic pain.

2 An exception is the Greatest Generation adults, who experienced no significant pain increases in most sites. This could be an artifact of mortality selection because the number of respondents in this generation dwindled to 0 by the 2017 and 2018 survey years. We can assume that between 2002 and 2016, the Greatest Generation sample was increasingly shaped by selective mortality (Zajacova and Burgard 2013), so that the remaining members of this cohort will be more and more advantaged and healthier than the original cohort.

3 While hypertension is dubbed the “silent killer” because it causes no pain or other noticeable symptoms (WHO 2013), adults with pain may be more likely to visit healthcare providers who can diagnose hypertension. Adults with hypertension may also be more likely to have comorbidities that cause pain.

4 The population aged 25–84 was 223.29 million in 2018. If the proportion with pain remained at 2002 levels (49.1%), 109.63 million adults would report pain. Instead, 120.13 million individuals (53.8%) reported pain in 2018, corresponding to 10.5 million more people.
