# Supplementary material for: Pain Trends Among American Adults, 2002–2018: Patterns, Disparities, and Correlates
Source: Demography. Author manuscript; Available in PMC 2021 Oct 1. (PMC8035485; doi:10.1215/00703370-8977691)
Supplement: Online Appendix [file NIHMS1683618-supplement-Online_Appendix.pdf]

ONLINE APPENDIX.

Robustness checks. In addition to the analytic steps outlined above, we did extensive additional work to confirm the reliability and validity of the results.

1) We estimated models of pain with time specified as quadratic and cubic; we also explored regression discontinuities by examining trends in earlier and later years separately. Results showed that a linear specification fit the data well: the higher-order terms were not statistically significant in most models, and the regression discontinuities results varied across population subgroups and the years where we divided the model but overall showed that the improved model fit in some cases would not be worth the additional complexities in modeling the trend.
2) Alternative specification of select covariates: We considered alternative specification of covariates, such as BMI categorized using 6 levels in order to capture possible nonlinearities (Preston, Mehta and Stokes 2013; Zajacova and Burgard 2012), and more detailed separation of categories within marital status, employment status, home ownership, and the number of children in the home. The different covariate specifications had no substantive impact on the findings relevant to pain trends.
3) Tighter definition of the analytic sample: the exclusion of proxy responses, foreign-born, and non-English speakers yielded generally comparable but steeper pain increases as might be evident from the stratified analyses in Table 3.
4) Alternative specification of the link function: we re-estimated regression models using linear probability and log-binomial models, as well as a Poisson model with robust standard errors where the log-binomial models would not converge (McNutt et al. 2003).
5) Diagnostics for the fully-adjusted models in Table 5: We checked that collinearity was not a problem in these models (we re-estimated using LPM without sampling-design adjustment on a randomly-selected multiply-imputed data file in order to calculate the variance inflation factor). The additive models shown have no collinearity issues, with the highest VIF for any covariate in either age model 2.5; the interaction models, unsurprisingly, indicated excessive collinearity pertaining to the time trend.
6) The NHIS is designed to be nationally representative for each year of data collection. We therefore re-estimated our models of trends using the year of interview rather than the more detailed year and month information. The results were nearly identical to those shown here.

Appendix Table 1. Pain prevalence and age-standardized pain prevalence among US adults aged 25-84, 2002 to 2018.

	Any pain	Joint	Back	Neck	Migraine	Facial
**A. Prevalence**						
2002	49.1	26.9	27.4	14.8	15.0	4.7
2003	51.0	29.0	28.7	15.6	15.1	4.5
2004	50.7	29.2	28.3	15.7	15.1	4.3
2005	50.9	29.2	29.7	15.7	14.9	4.6
2006	49.7	27.7	28.8	15.6	15.1	4.7
2007	47.0	26.4	26.7	14.0	12.5	4.1
2008	50.3	29.3	28.3	14.8	13.3	4.5
2009	53.4	30.7	29.9	16.4	15.9	5.3
2010	53.5	31.4	30.2	17.0	16.2	5.0
2011	53.6	32.1	30.6	16.7	16.4	5.1
2012	50.8	28.9	29.3	15.5	13.8	4.9
2013	52.1	30.6	30.5	16.2	15.6	4.7
2014	52.4	30.9	29.8	15.9	14.8	4.7
2015	54.4	32.3	31.8	17.3	15.3	4.3
2016	53.6	32.2	30.4	16.3	15.2	4.8
2017	53.5	32.3	30.2	16.4	15.0	4.4
2018	53.8	32.5	31.4	17.2	15.6	5.3
**B. Age-standardized prevalence**						
2002	49.4	27.7	27.5	14.9	14.6	4.7
2003	51.5	29.9	28.9	15.8	14.9	4.5
2004	51.1	29.9	28.5	15.8	14.9	4.3
2005	51.2	29.9	29.8	15.8	14.6	4.6
2006	50.1	28.3	29.0	15.7	15.0	4.7
2007	47.2	26.7	26.7	14.0	12.3	4.1
2008	50.5	29.6	28.4	14.8	13.2	4.4
2009	53.4	30.8	29.9	16.5	15.8	5.3
2010	53.6	31.5	30.3	17.0	16.2	5.0
2011	53.5	32.0	30.6	16.7	16.4	5.1
2012	50.7	28.6	29.3	15.5	13.9	4.9
2013	51.8	30.1	30.4	16.2	15.8	4.7
2014	52.1	30.3	29.7	15.9	15.1	4.7
2015	54.0	31.7	31.6	17.3	15.5	4.3
2016	53.2	31.5	30.2	16.3	15.6	4.9
2017	53.0	31.5	30.1	16.4	15.5	4.5
2018	53.2	31.5	31.1	17.1	16.1	5.4

Source: NHIS 2002-2018. Panel A shows weighted proportions in the total sample.
Panel B shows weighted age-standardized proportions using the 2010 US population age structure.

Appendix Figure 1. Pain Trend 2002-2018 by Income and Age, US Adults Age 25-84

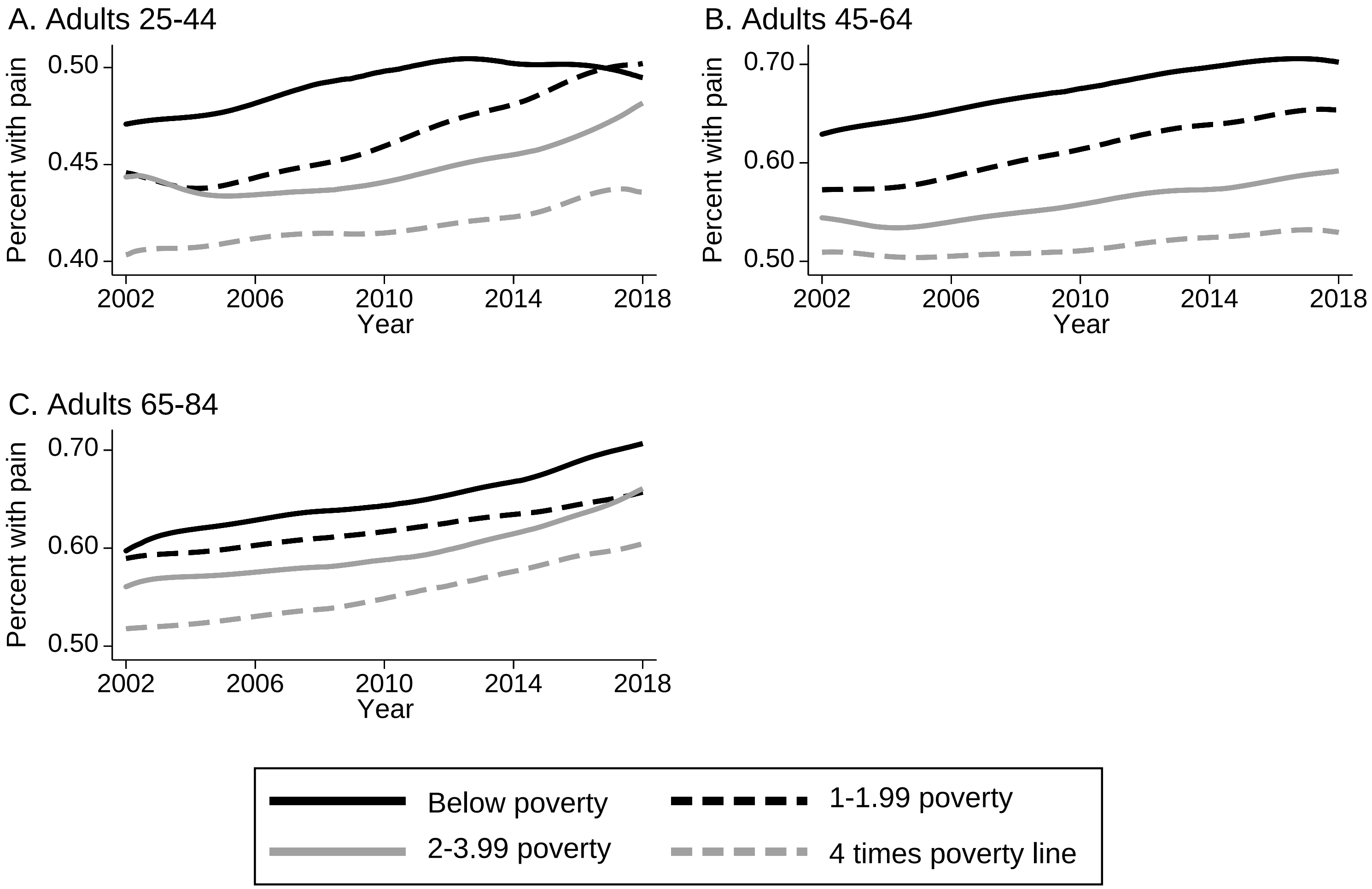

From semiparametric age- and income-stratified, demographics-adjusted logistic model of 'any pain.'
